# Cherubism, emphasizing diagnosis, therapeutic management strategies, and outcomes: a case series

**DOI:** 10.1186/s13256-026-06184-8

**Published:** 2026-05-30

**Authors:** Pouyan Aminishakib, Seyed MohammadMoein Hosseini, Farnoosh Mohammadi, Marjan Yaghmaie, Alireza Modarresi, Hossein Pashaiefar, Saba Sadat Tabatabaei

**Affiliations:** 1 Department of Oral and Maxillofacial Pathology, School of Dentistry, Tehran University of Medical Sciences, Tehran, Iran; 2 School of Dentistry, Tehran University of Medical Sciences, Tehran, Iran; 3 Department of Oral and Maxillofacial Surgery, School of Dentistry, Tehran University of Medical Sciences, Tehran, Iran; 4 Hematology, Oncology & Stem Cell Transplantation Research Center, Tehran University of Medical Sciences, Tehran, Iran; 5 Department of Oral and Maxillofacial Surgery, School of Dentistry, Islamic Azad University Tehran Medical Sciences, Tehran, Iran

**Keywords:** Cherubism, Maxillofacial surgery, SH_3_-BP_2_, Case series, Denosumab, Calcitonin

## Abstract

**Background:**

Cherubism is a rare fibro-osseous disorder that shows autosomal dominant inheritance, primarily caused by SH_3_-BP_2_ mutations. This study presents nine cases with a primary focus on therapeutic strategies and outcomes, including the use of denosumab in a refractory case. Genetic analysis was performed in a subset of patients, and a practical diagnostic and therapeutic algorithm is proposed.

**Case presentation:**

Nine Persian patients (7 males, 2 females; onset 2–14 years), are reported with clinical, radiographic, histopathological, and genetic findings (in three cases) with identified mutations in two cases. Treatment strategies included observation, intra-lesional corticosteroid, surgery, denosumab, and calcitonin. Outcomes varied by modality and timing, with denosumab and calcitonin proving effective in controlling progression after surgical relapse in one case.

**Conclusion:**

This case series highlights the variable natural behavior of cherubism and provided an algorithm for diagnosis and management based on expert consensus.

## Background

Cherubism is a rare fibro-osseous skeletal disorder characterized by symmetrical enlargement of the jaws. In some cases, orbital involvement may occur due to extension of the lesion from the maxilla [1], resulting in upward gaze deviation [2]. The disease is primarily hereditary, following an autosomal dominant inheritance pattern, and is associated with mutations in the SH_3_-BP_2_ (SH_3_-binding protein _2_) gene located on chromosome 4p16.3 [3, 4]. The association of cherubism with several genetic syndromes was reported, including Noonan syndrome, Ramon syndrome, fragile X syndrome, and neurofibromatosis type 1 [5]. Notably, Noonan syndrome was considered as a differential diagnosis in cases lacking SH_3_-BP_2_ mutations but presenting with PTPN11 (Protein Tyrosine Phosphatase Non-Receptor Type 11) mutation [6].This highlights the importance of comprehensive genetic testing in atypical cases.

Cherubism typically manifests in early childhood, usually between the ages of 2 and 7 years, the lesions in the jaws gradually enlarging until puberty [7]. After this period, often regress and remodel, resulting in the affected bone structures returning to a more normal configuration during adulthood [8].

The severity of cherubism varies widely and is classified into three clinical subtypes: non-invasive, can be observed in adolescents with little or no progression; aggressive, predominantly affecting young children and characterized by rapid lesion expansion; and a silent subtype, which is mainly observed in older adults, with the stable lesions [9]. Beyond these subtypes, a grading system has been proposed to classify cherubism based on location and severity:

- Grade I: lesions confined to the mandible, without root resorption.
- Grade II: involvement of both mandible and maxilla, without root resorption.
- Grade III: aggressive mandibular lesions with root resorption.
- Grade IV: aggressive lesions in both mandible and maxilla, with root resorption.
- Grade V: adolescent cases with very aggressive lesions affecting both jaws.
- Grade VI: similar to Grade V, but with orbital involvement [10, 11].

Cherubism can lead to significant dental complications [12], and in severe cases, the condition may cause airway obstruction.

The hallmark histopathological pattern is characterized multinucleated giant cells scattered within a fibrous connective tissue stroma, and demonstrating positivity for Tartrate-Resistant Acid Phosphatase (TRAP), to confirm their osteoclastic activity [13].

Management strategies depend on disease severity; mild to moderate cases are generally managed conservatively, as the lesions often resolve spontaneously with time. Surgical intervention is usually reserved for severe cases, particularly when functional or aesthetic concerns are present, and is ideally performed after skeletal growth has ceased. However, in some patients, lesion progression remains difficult to control despite intervention [14] In addition to these approaches, interventional and non-surgical methods are also employed. These include intra-lesional corticosteroid injections, the administration of inhaled calcitonin, and denosumab injections.

The primary objective of this study was to describe the clinical, histopathological, and genetic features of cherubism in nine Persian patients, with particular emphasis on diagnostic and therapeutic management strategies. Special attention was given to the role of denosumab and calcitonin in a case with postsurgical relapses. Based on expert experiences, we present a hypothesis-generating, experience-based algorithm, given the absence of universally accepted, standardized guidelines for the management of cherubism, especially in pediatric and refractory cases, such experience-based frameworks may offer preliminary clinical guidance to help clinicians in managing this rare condition.

## Study design

Patients were identified by retrospective review of their records. Inclusion criteria were: (1) clinical presentation consistent with cherubism (symmetrical, painless swelling of the posterior mandible and/or maxilla with onset in childhood); (2) compatible radiographic findings on panoramic radiograph and/or Cone Beam Computed Tomography (CBCT)/Computed Tomography (CT); (3) availability of baseline clinical records and imaging; (4) availability of histopathological specimen and definitive diagnosis of cherubism; and (5) written informed consent for use of clinical data and images. Exclusion criteria were (1) insufficient clinical or radiographic data; (2) alternative diagnoses more likely than cherubism; (3) prior surgical treatment that precluded assessment of baseline lesions; (4) or refusal of consent. Specific information was de-identified to protect patient privacy.

Genetic testing was performed selectively in three patients (Cases 1, 2, and 3) due to logistical constraints (cost and patient consent) and to include a representative sample of both sporadic (Cases 1 and 2) and familial (Case 3) presentations. This selective approach allowed us to evaluate the contribution of SH_3_-BP_2_ mutations, while acknowledging that comprehensive testing of all patients was not feasible in this study.

Genomic DNA was extracted from peripheral blood leukocytes of the three patients using a standard salting-out method. DNA concentration and purity were assessed using a NanoDrop spectrophotometer (Thermo Fisher Scientific, USA) and confirmed by agarose gel electrophoresis. For mutation analysis, exon 9 of the SH_3_-BP_2_ gene, which harbors the mutational hotspot associated with cherubism, was amplified by Polymerase Chain Reaction (PCR) using specific primers (forward: 5’-TGAGCTTTTTAGGGTCACAGG-3’; reverse: 5’-GGCTTTACATGGTGCTGTGT-3’). PCR reactions were performed in a total volume of 25 µL containing 100 ng of genomic DNA, 10 pmol of each primer, 200 µM (Deoxynucleotide Triphosphates) dNTPs, 1.5 mM MgCl₂, and 1 U of Taq DNA polymerase (Thermo Fisher Scientific, USA). The cycling conditions were: initial denaturation at 95 °C for 5 min, followed by 35 cycles of denaturation at 95 °C for 30 s, annealing at 61 °C for 30 s, extension at 72 °C for 45 s, and a final extension at 72 °C for 7 min. The PCR products were purified using the [QIAquick PCR Purification Kit, Qiagen] and subjected to bi-directional Sanger sequencing using the ABI Prism 3500xl Genetic Analyzer (Applied Biosystems, USA). Sequencing chromatograms were analyzed with Chromas software (Technelysium Pty Ltd, Australia) and compared with the reference SH3BP2 sequence (GenBank accession no. NM_018957.6) using BLAST and Mutation Surveyor software (SoftGenetics, USA).

The diagnostic and treatment algorithm was developed through consensus process involving a multidisciplinary experts. This panel comprised: (1) Two oral and maxillofacial surgeons, one with a fellowship in head and neck oncology surgery and the other with a fellowship in craniofacial surgery. (2) Two oral and maxillofacial pathologists. (3) Two medical geneticists. (4) One pediatric oncologist/hematologist. Expert opinions were gathered through online/In-person consultation process. When consensus among all panel members could not be reached on a specific point, the final decision was deferred to the specialist in that particular field. The final algorithm was reviewed and approved by all participating members, this algorithm is based on expert consensus and clinical experience derived from the presented case series and does not represent an evidence-based guideline.

The study was approved by the Tehran University of Medical Sciences Institutional Review Board (IR.TUMS.AMIRALAM.REC.1403.010).

## Case series

**Fig. 1:**
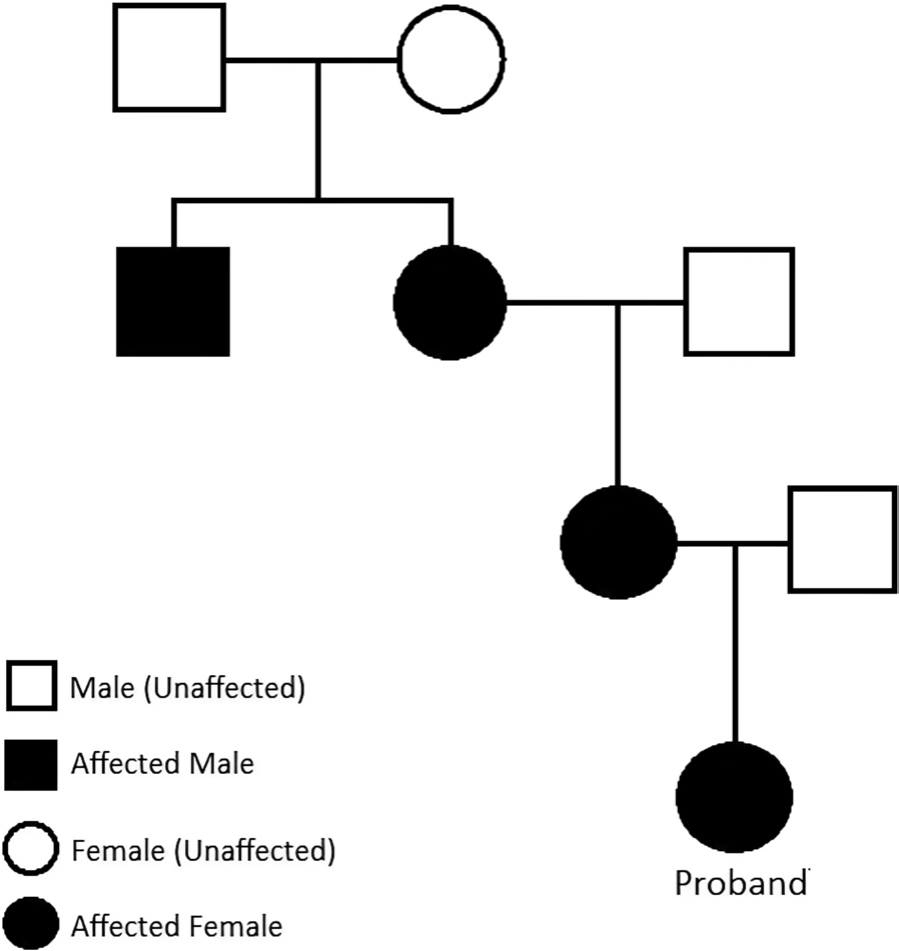
Pedigree of Case 3. Pattern of the disease could be seen in proband’s mother side

This case series included nine patients (Table 1), all Persian, with an age of onset ranging from 2 to 14 years, suggesting early childhood onset. There was a predominance of males. An inheritance pattern was identified in three patients: two brothers (Cases 8 and 9) and one female patient (Case 2), whose pedigree is shown in Fig. 1.

**Table 1:** Patients Data Summary

Case NO	Onset Age	Sex	Family history	Grading^*^	Involved Jaws	Treatment	Follow-up/Outcome	Genetic Test Results	Specific Clinic/Radio/Pathologic features
*1*	5	M	No	II	Man^1^ & Max^2^	Surgery (resection) (2 times) + Denosumab (17 months) + calcitonin nasal spray (3 months)	Recurrences noted after surgery (5 and 7 years old), no recurrance after treatment with Denosumab, but there were side effects, no relapse after the use of calcitonin nasal spray	Positive	Painless, Bilateral swelling
*2*	12	M	No	II	Man & Max	Observation until skeletal maturity	After 26 months, slow progression; no intervention required up to skeletal maturity	Negative	Painful, bilateral swelling, Unerupted tooth in affected area
*3*	6	F	Yes	II	Man & Max	Injections of Triamcinolone	After 26 months, no progression; no intervention required up to skeletal maturity	Positive	Painless, Bilateral swelling, Cortical perforation, Displacement of dental buds
*4*	4	M	No	V	Man, Max, Sphenoid & Temp^3^	Injections of Triamcinolone	After 155 months, Shrinkage of the lesion is noted	Not performed	Bilateral swelling
*5*	11	M	No	I	Man	Injections of Triamcinolone	After 36 months, Shrinkage of the lesion is noted	Not performed	Bilateral swelling, No perforation, Displacement of dental buds, Perivascular hyalinization
*6*	2	F	No	I	Man	Injections of Triamcinolone + Surgery (resection)	After 33 months, no progression is noted	Not performed	Bilateral swelling, on ultrasound, a hypo-echoic lesion was noted with no lymphadenopathy
*7*	14	M	No	I	Man	Not available	Not available	Not performed	Bilateral swelling, Coronoid processes affected, Displacement and Impaction of tooth
*8*	6	M	Yes	I	Man	Injections of Triamcinolone	After 108 months, shrinkage of the lesion is noted	Not performed	Bilateral swelling
*9*	8	M	Yes	I	Man	Observation until skeletal maturity	After 26 months, slow progression; no intervention required up to skeletal maturity	Not performed	Bilateral swelling

*The grading system is based on [Papadaki ME et al.], [2012], Not available: Data not available due to retrospective design or incomplete records

Not performed: Test was not conducted due to clinical or logistical limitations

**Fig. 2:**
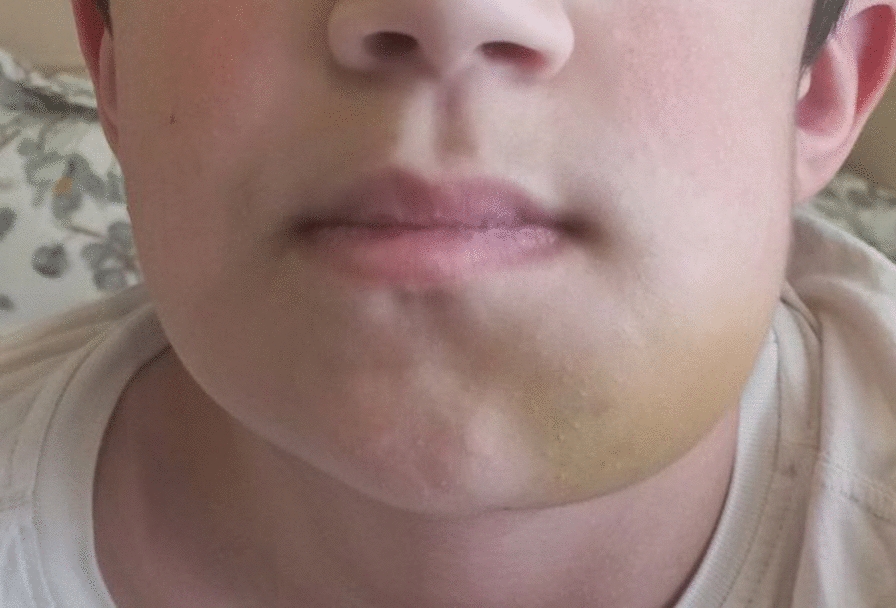
Facial profile of Case 2. A bilateral pattern is observed in the patient, but the lesion is larger on the left side

The most notable clinico-radiopathologic features included bilateral jaw swelling, (sometimes asymmetrical, as shown in Fig. 2), displacement and impaction of teeth, and perivascular hyalinization. Clinically, all patients presented with progressive swelling of the jaws, most commonly affecting the mandible. Swelling was often bilateral and symmetrical, although asymmetry was observed in some older patients. The disease typically developed in early childhood, progressed gradually, and usually stabilized or regressed after puberty; however, relapse after surgery was noted in one case. Delayed eruption of teeth was frequently observed. Pain was generally absent, with only occasional mild discomfort reported. In some cases, a positive family history supported the hereditary nature of the disease.

**Fig. 3:**
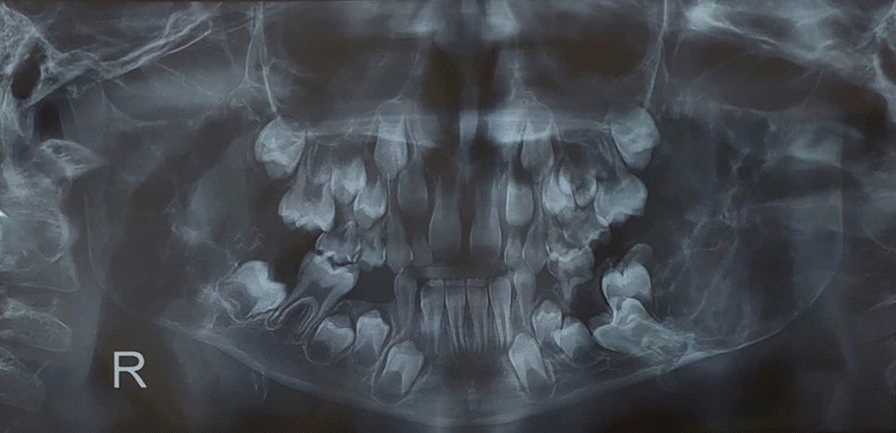
Panoramic radiograph from case 8 demonstrating bilateral multilocular radiolucent lesions involving the posterior mandible, with thinning and expansion of cortical plates and displacement of developing teeth, consistent with cherubism

Radiographic evaluation revealed bilateral, multilocular radiolucent lesions primarily affecting the mandible, with occasional maxillary and involvement of the coronoid process. The lesions caused cortical expansion, thinning, and in some cases, perforation. In several patients, imaging demonstrated cystic changes with a coarse trabecular pattern and expansile remodeling of the affected bone. No evidence of malignancy, root resorption, or condylar involvement was identified (Fig. 3, Case 8).

**Fig. 4:**
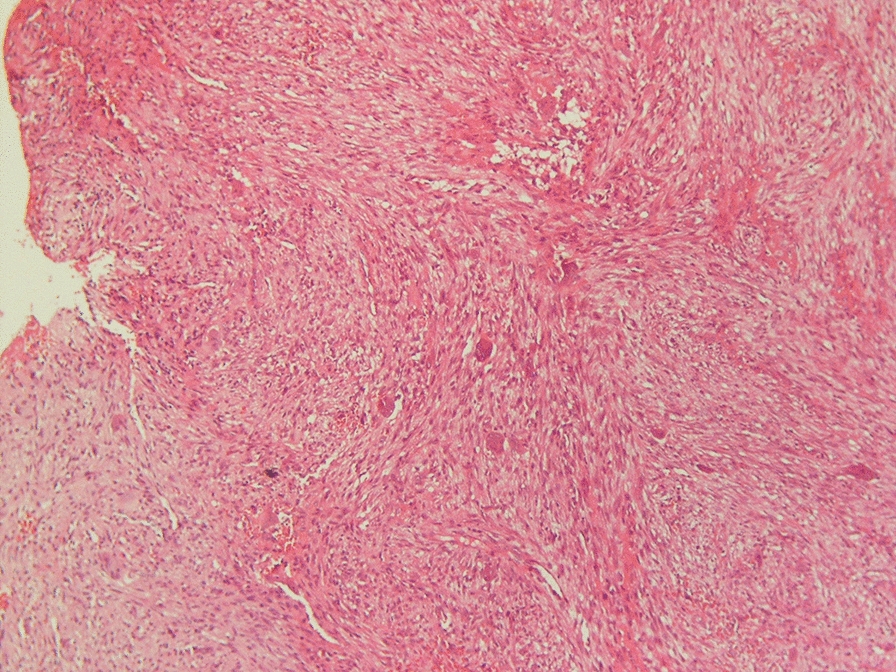
Histopathological section from Case 6, showing fibrovascular connective tissue stroma with spindle-shaped fibroblasts arranged in a whorled pattern, intermixed with multinucleated giant cells. (H&E stain, × 100)

Histopathological examination revealed a fibrovascular stroma containing numerous multinucleated giant cells. These giant cells were scattered throughout the fibrous tissue and frequently located adjacent to irregular trabeculae of newly formed woven bone. No evidence of atypical mitosis or malignancy was observed. These microscopic findings are consistent with the characteristic histopathology of cherubism (Fig. 4, Case 6).

Genetic analysis of exon 9 of the SH_3_-BP_2_ gene revealed mutations in two of the three tested patients (Cases 1 and 3). In case 1, genetic alteration was detected in exon 9 of SH_3_-BP_2_ gene: NM_001122681.2(SH_3_-BP_2_):c.1252C > A (p.Pro418Thr), with germline classification of Pathogenic/likely pathogenic. In Case 3 the alteration was detected in exon 9 of SH_3_-BP_2_ gene: NM_001122681.2(SH_3_-BP_2_):c.1253C > A (p.Pro418His) with classification of Pathogenic. In case 2, no reportable genetic alterations were identified within the exon 9 of SH_3_-BP_2_ gene, underscoring that the absence of SH3BP2 variants does not exclude the diagnosis of cherubism.

Treatment decisions in the cases were individualized based on each patient’s condition (Table 1). Conservative management (observation or intra-lesional corticosteroids) was employed in seven patients and resulted in lesion stabilization or regression in all cases. Surgical intervention (resection) in Case 1 led to recurrences at ages 5 and 7 years-old, Following these recurrences, a pediatric hematologist-oncologist, prescribed denosumab (Xgeva®) at a dose of 120 mg, administered every two weeks, for 14 months. The patient experienced severe pain in the spine and lower extremities, occasionally accompanied by urinary incontinence. He was subsequently referred to a multidisciplinary medical commission composed of multiple specialists (oral and maxillofacial surgeons, Pediatrician, Pediatric Hematologist-Oncologist, and Otolaryngologist). During the commission’s review, with a diagnosis of cherubism for the patient, treatment plan transitioned to continue denosumab injection with the daily use of calcitonin nasal spray at a dose of 200 IU. The injections were discontinued owing to financial constraints; nevertheless, treatment with calcitonin was commenced and subsequently continued. After three months commencing calcitonin therapy, clinical examinations show no significant growth in the patient’s jaws, but the patient experienced severe nausea, which was present both during meals and in the fasting state. The treatment plans and outcomes for the majority of other patients primarily involved conservative management strategies, and only 2 patients received surgical intervention (resection). These plans included active observation through regular, periodic patient visits (based on patient’s condition from 1 month interval to 3 months) and intra-lesional injections of triamcinolone (TRIAMHEXAL, 40 mg/mL), the frequency of these injections varied based on individual patient conditions, ranging from every two weeks to every three months, Moreover, the injection frequency for each patient varied across different time periods; when a slower lesion growth rate was observed, the interval between injections was extended, in some cases up to three months. In most patients, a reduction in lesion growth, cessation of growth, or even lesion shrinkage was observed.

The duration of all treatment plans was determined based on individual patient conditions, including factors such as lesion size, rate of regression, or growth and other factors. Treatment was continued as long as a reduction in lesion growth rate, shrinkage, or lack of progression was observed. The treatment course was concluded at the surgeon’s discretion when therapeutic indications were no longer present during patient examination (e.g., sufficient lesion reduction and skeletal maturity). Resistance to the treatment, or adverse side effects necessitated a modification of the treatment plans.

Statistical analysis was not feasible due to the small sample size, some observable patterns were noted. Patients with earlier onset (≤ 6 years) are more likely to present with more extensive disease involvement (higher grading). Conservative management strategies (observation or intra-lesional corticosteroids) were associated with stabilization or regression in the majority of cases (7 out 9 patients); and early surgical intervention was followed by relapse. These findings suggest a potential association between treatment modality and clinical outcome; however, given the descriptive nature, these observations should be interpreted with caution, and further investigation is needed.

## Discussion

In this article, we report nine cases. The findings provide insights that may aid in the diagnosis and management of cherubism, a rare disorder of the jaw bones, with approximately 300 cases reported worldwide as of 2012 [9].

However, given the small number of patients, the potential for selection bias of them, and the variability among them, not all cases underwent uniform genetic or radiological evaluation, resulting in dataset heterogeneity, which may limit the interpretation of genotype–phenotype correlations, also there was a lack of standardization in both diagnostic work-up and treatment protocols, as management decisions were individualized based on patient-specific factors and resource availability,. Moreover, due to the case-series design, this study is primarily descriptive in nature. Consequently, causal inferences cannot be drawn, and statistical analyses remains limited. These limitations highlight the need for larger, prospective, and standardized studies to better define optimal management strategies. The proposed algorithm is based on a small, heterogeneous sample (n = 9) without external validation and should be interpreted as an experience-based framework rather than a definitive guideline.

The clinical features of cherubism, first described in 1933 [15], are distinctive and have remained consistent, these include upward gaze, pronounced (usually bilateral) facial swelling with firm bony consistency, and absence of pain [16]. While all our cases presented with bilateral involvement, researchers have reported rare unilateral cases [17, 18]. Therefore, diagnosis requires clinico-radiopathologic correlation. Radiographic findings typically include a “floating tooth” appearance, tooth displacement, cortical expansion, and absence of root resorption [19, 20]; presented cases demonstrated these similar features. On computed tomography (CT) and magnetic resonance imaging (MRI), lesions often exhibit a soap bubble–like remodeling pattern, though CT is the preferred imaging modality [21]. Histologically, a hallmark features is perivascular cuffing of eosinophilic collagen around blood vessels [17]. Differential diagnoses include brown tumor, central giant cell granuloma (CGCG), fibrous dysplasia, and giant cell tumor [9, 22, 23]. Histological findings in our cases were consistent with these established descriptions.

The SH_3_-BP_2_ gene has been implicated as a primary factor in this condition [24, 25]. This gene encodes an adaptor protein involved in signaling pathways that regulate osteoclast differentiation and inflammatory response; gain-of-function alterations in SH_3_-BP_2_ lead to enhanced NF-κB activation and increased osteoclastogenesis [26]. Despite this role, its precise contribution to cherubism remains unclear due to conflicting findings and the reported occurrence of non-familial cases [27]; also while SH_3_-BP_2_’s effects are recognized in Wolf-Hirschhorn syndrome [28], this syndrome is distinct from cherubism. Notably, in this case series, one patient diagnosed with cherubism via clinico-radiopathological examination, tested negative for mutations in this gene, underscoring that genetic testing is supplementary, especially in complex cases, and negative result does not definitively exclude the disease.

Treatment plans in our series were individualized according to disease severity, patient age, functional/aesthetic considerations, and financial constraints. The self-limiting nature of cherubism after puberty is supported by the favorable outcomes observed in patients managed conservatively including “wait and see” and intra-lesional corticosteroid approaches (cases 2, 3, 4, 5, 8, and 9) as reported in previous studies [29, 30], and relapses following early surgical interventions in Case 1, suggest avoiding surgery before skeletal maturity, unless the condition is life-threatening, such as in cases with a risk of airway obstruction. When necessary, reconstructive procedures [31] and dental implants [32] may be used to restore proper occlusion in patients with affected tooth buds.

Given that denosumab exerts its effects by inhibiting RANKL and subsequently reducing osteoclast activity [33], its off-label use has been explored in cherubism [34], particularly in cases where reducing the need for surgical intervention or preventing postoperative recurrence is desirable [35]. In our series, denosumab was administered in one patient as part of the treatment strategy. However, careful monitoring is essential during treatment, particularly with regard to electrolyte balance and this treatment carries significant risks in children, including rebound hypercalcemia and disruption of calcium and phosphate homeostasis [36]. In our case, although disease stabilization was achieved after denosumab administration, the patient experienced notable systemic symptoms, and treatment discontinuation was influenced by financial constraints.

Calcitonin has also been investigated as a conservative anti-resorptive agent in the management of cherubism, with treatment outcomes ranging from disease stabilization to partial regression of jaw lesions [37, 38]. Its mechanism is based on the inhibition of osteoclastic bone resorption, making it a theoretically attractive option for controlling lesion progression [39], However, the evidence remains limited and heterogeneous, and no standardized treatment protocol has been established. In the present series, although calcitonin was not part of the initial therapeutic protocol, its later use in a recurrent and medically complex case was associated with short-term clinical stabilization; however, significant adverse effects was noted, particularly persistent nausea. These findings, together with previous reports, suggest that while calcitonin may offer a non-invasive therapeutic alternative in selected cases, its clinical utility is constrained by inconsistent efficacy and tolerability, underscoring the need for further controlled studies [40].

**Fig. 5:**
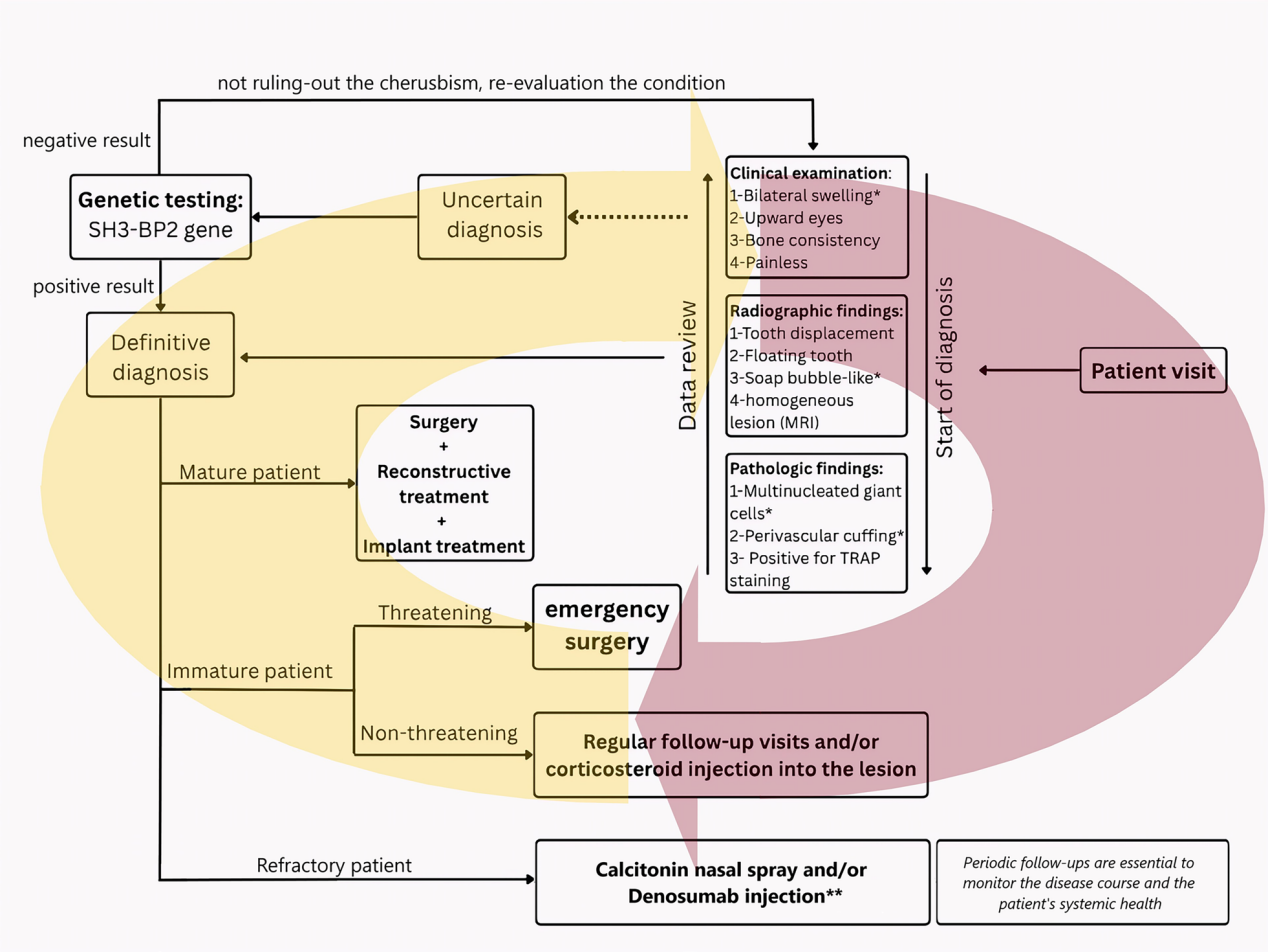
Diagnostic and therapeutic algorithm. *Key features in each section relevant for diagnosis decision-making. **Attention must be paid to the potential adverse effects throughout the course of therapy. Red and yellow arrows indicating the importance of follow-up visits and re-evaluating the patient’s condition, which may modify the treatment plan during the course of therapy, based on the new outcomes

To facilitate the diagnosis and treatment process for cherubism, this article introduces an algorithm. The proposed diagnostic and therapeutic algorithm (Fig. 5) is directly supported by the outcomes observed in this study, supplemented through expert panel sessions, and the collective experience of specialists. Based on this diagram, we start diagnostic approaches with clinical data, followed by radiographic and pathological evaluation, after gathering all data and reviewing them in a multi-disciplinary insight we can make a disicion; which leads us to a definitive or uncertain diagnosis. Genetic test, as a supplementary test can help us to diagnose the condition. Also the treatment is planned based on the patient’s condition. The proposed algorithm is not evidence-based and is derived from expert consensus and clinical experience.

## Conclusion

As a self-limiting disease, cherubism is not usually a cause for concern, except in cases where it threatens life due to the possibility of damage to the respiratory system or vision. The majority of patients are children, and due to the appearance of the disease, it usually causes great concern for parents. Individual patient responses to therapeutic interventions vary. In cases of refractory patients, the consideration of denosumab and/or calcitonin may be warranted. However, careful attention must be paid to their potential side effects, and continuous follow-ups are essential to monitor the patient's systemic status alongside the progression of the lesion. The patient and the family should be reassured, and an appropriate diagnosis and treatment plan should be selected to prevent any further problems. The key takeaway from this case series is that genetic testing is helpful but not definitive; follow-up is the mainstay; surgery is reserved for severe cases.

## Data Availability

The datasets generated and/or analysed during the current study are not publicly available due to patient privacy and confidentiality restrictions but are available from the corresponding author on reasonable request.

## Abbreviations

CBCT: Cone beam computed tomography
CT: Computed tomography
MRI: Magnetic resonance imaging
PCR: Polymerase chain reaction
dNTPs: Deoxynucleotide triphosphates
TRAP: Tartrate-resistant acid phosphatase
PTPN11: Protein tyrosine phosphatase non-receptor type 11
CGCG: Central giant cell granuloma

## References

[1] Irfan S, Cassels-Brown A, Hayward JM, Corrigan AM. Orbital cherubism. Orbit. 1997;16:109–12.

[2] Emoto Y, Emoto H, Fujie W, Wakakura M, Yamaguchi A, Sugiura H, *et al*. Uncorrectable oblique astigmatism and impaired binocular vision in case of orbital cherubism. Neuroophthalmology. 2007;31:191–5.

[3] Li CY, Yu SF. A novel mutation in the SH3BP2 gene causes cherubism: case report. BMC Med Genet. 2006;7: 84. 17147794 10.1186/1471-2350-7-84 PMC1764878

[4] Tiziani V, Reichenberger E, Buzzo CL, Niazi S, Fukai N, Stiller M, *et al*. The gene for cherubism maps to chromosome 4p16. Am J Hum Genet. 1999;65:158–66. 10364528 10.1086/302456 PMC1378086

[5] van Capelle CI, Hogeman PHG, van der Sijs-Bos CJM, Heggelman BGF, Idowu B, Slootweg PJ, *et al*. Neurofibromatosis presenting with a cherubism phenotype. Eur J Pediatr. 2007;166:905–9. 17120035 10.1007/s00431-006-0334-6

[6] Jafarov T, Ferimazova N, Reichenberger E. Noonan-like syndrome mutations in PTPN11 in patients diagnosed with cherubism. Clin Genet. 2005;68:190–5. 15996221 10.1111/j.1399-0004.2005.00475.x

[7] Carroll AL, Sullivan TJ. Orbital involvement in cherubism. Clin Exp Ophthalmol. 2001;29:38–40. 11272784 10.1046/j.1442-9071.2001.00363.x

[8] Hitomi G, Nishide N, Mitsui K. Cherubism: diagnostic imaging and review of the literature in Japan. Oral Surg Oral Med Oral Pathol Oral Radiol Endod. 1999;87:228–33. 10.1016/s1079-2104(96)80060-6 8734715

[9] Papadaki ME, Lietman SA, Levine MA, Olsen BR, Kaban LB, Reichenberger EJ. Cherubism: best clinical practice. Orphanet J Rare Dis. 2012;7: S6. 22640403 10.1186/1750-1172-7-S1-S6 PMC3359956

[10] Raposo-Amaral CE, Guidi MC, Warren SM, Almeida AB, Amstalden EMI, Tiziani V, *et al*. Two-stage surgical treatment of severe cherubism. J Craniofac Surg. 2007;18:1091–8. 17522488 10.1097/01.sap.0000248141.36904.19

[11] Kalantar Motamedi MH. Treatment of cherubism with locally aggressive behavior presenting in adulthood: report of four cases and a proposed new grading system. J Oral Maxillofac Surg. 2009;67:1012–9. 10.1016/s0278-2391(98)90618-8 9820222

[12] AlAli AM, Dashti H, Al-Yahya Y, Ali H. A report of two atypical genetic cases of cherubism: reduced penetrance and sporadic occurrence. J Oral Maxillofac Surg Med Pathol. 2021;33:234–8.

[13] Friedrich RE, Scheuer HA, Zustin J, Grob T. Cherubism: a case report with surgical intervention. Anticancer Res. 2016;36:3109–12. 27272835

[14] Peñarrocha M, Bonet J, Mínguez JM, Bagán JV, Vera F, Mínguez I. Cherubism: a clinical, radiographic, and histopathologic comparison of 7 cases. J Oral Maxillofac Surg. 1999;57:530–5. 10.1016/j.joms.2006.02.003 16713807

[15] Jones WA. Familial multilocular cystic disease of the jaws. Am J Cancer. 1933;17:946–50.

[16] Lima GM, Almeida JD, Cabral LA. Cherubism: clinicoradiographic features and treatment. J Oral Maxillofac Res. 2010;1: e2. 10.5037/jomr.2010.1202. 24421967 10.5037/jomr.2010.1202 PMC3886048

[17] Johnston DT, Hudson JW, Wells NG, Pickup JD. True unilateral mandibular cherubism: a literature review and case report. J Oral Maxillofac Surg. 2020;78:228–34. 10.1016/j.joms.2019.08.027. 31655027 10.1016/j.joms.2019.08.027

[18] Reade PC, McKellar GM, Radden BG. Unilateral mandibular cherubism: brief review and case report. Br J Oral Maxillofac Surg. 1984;22:189–94. 10.1016/0266-4356(84)90096-2. 6234934 10.1016/0266-4356(84)90096-2

[19] Caballero R, Vinals H. Cherubism: a study of three generations. Med Oral. 1998;3:163–71. 11507493

[20] Gomes MF, de Souza Setúbal Destro MF, de Freitas Banzi EC, dos Santos SH, Claro FA, de Oliveira Nogueira T. Aggressive behaviour of cherubism in a teenager: 4-years of clinical follow-up associated with radiographic and histological features. Dentomaxillofac Radiol. 2005;34(5):313–8. 10.1259/dmfr/32866350. 16120883 10.1259/dmfr/32866350

[21] Beaman FD, Bancroft LW, Peterson JJ, Kransdorf MJ, Murphey MD, Menke DM. Imaging characteristics of cherubism. AJR Am J Roentgenol. 2004;182(4):1051–4. 10.2214/ajr.182.4.1821051. 15039186 10.2214/ajr.182.4.1821051

[22] Rajashekarappa M, Aroor R, Parameshwarappa KP, Sujata M, Shetty A. Cherubism: a rare case in childhood. J Dermatol Sci. 2024;125:e27–30. 10.1016/j.jdermsci.2024.07.002.

[23] Misra SR, Mishra L, Mohanty N, Mohanty S. Cherubism with multiple dental abnormalities: a rare presentation. BMJ Case Rep. 2014;2014: bcr2014206721. 10.1136/bcr-2014-206721. 25301429 10.1136/bcr-2014-206721 PMC4195220

[24] Kueper J, Tsimbal C, Olsen BR, Kaban L, Liao EC. SH3BP2-related fibro-osseous disorders of the maxilla and mandible: a systematic review. Int J Oral Maxillofac Surg. 2022;51(1):54–61. 33941395 10.1016/j.ijom.2021.04.001

[25] Chrcanovic BR, Guimarães LM, Gomes CC, Gomez RS. Cherubism: a systematic literature review of clinical and molecular aspects. Int J Oral Maxillofac Surg. 2021;50(1):43–53. 32620450 10.1016/j.ijom.2020.05.021

[26] Kittaka M, Yoshimoto T, Hoffman H, Levitan ME, Ueki Y. RANKL-independent osteoclastogenesis in the SH3BP2 cherubism mice. Bone Rep. 2020;12: 100258. 10.1016/j.bonr.2020.100258. 32258251 10.1016/j.bonr.2020.100258 PMC7118294

[27] Deshmukh R, Joshi S, Deo PN. Nonfamilial cherubism: a case report and review of literature. J Oral Maxillofac Pathol. 2017;22(1):181. 10.4103/0973-029X.203791 PMC5406808 28479714

[28] Lietman SA, Yin L, Levine MA. SH3BP2 mutations potentiate osteoclastogenesis via PLCγ. J Orthop Res. 2010;28(11):1425–30. 20872577 10.1002/jor.21164 PMC2948751

[29] Benwadih S, Derdabi A, Dani B, Boulaadashas M. Cherubism: About a sporadic case. Case Rep Surg. 2025;7:B.142. Available from: https://www.casereportsofsurgery.com/archives/2025.v7.i1.B.142

[30] Sidorowicz W, Kubasiewicz-Ross P, Dominiak M. Familial cherubism: clinical and radiological features. Case report and review of the literature. Eur J Paediatr Dent. 2018;19(3):213–7. 30063153 10.23804/ejpd.2018.19.03.8

[31] Gomes MF, Diniz MG, Silva TA, Gomez RS. Clinical and surgical management of an aggressive cherubism treated with autogenous bone graft and calcitonin. ISRN Dent. 2011;2011: 340960. 10.5402/2011/340960. 21991467 10.5402/2011/340960 PMC3169853

[32] Dewan K, Bishop K. Management of a patient suffering with cherubism with dental implants. Eur J Prosthodont Restor Dent. 2011;19:89–92. 21780729

[33] Hanley DA, Adachi JD, Bell A, Brown V. Denosumab: mechanism of action and clinical outcomes. Int J Clin Pract. 2012;66(12):1139–46. 10.1111/ijcp.12022. 22967310 10.1111/ijcp.12022 PMC3549483

[34] Bar Droma E, Beck-Rosen G, Ilgiyaev A, Fruchtman Y, Abramovitch-Dahan C, Levaot N, *et al*. Positive outcomes of Denosumab treatment in 2 patients with cherubism. J Oral Maxillofac Surg. 2020;78(12):2226–34. 10.1016/j.joms.2020.06.016. 32649899 10.1016/j.joms.2020.06.016

[35] Liles SI, Hoppe CI, Arnold L. Denosumab therapy in cherubism. Cleft Palate Craniofac J. 2023;60(12):1665–73. 10.1177/10556656221113891. 35821585 10.1177/10556656221113891

[36] Boyce AM. Denosumab: an emerging therapy in pediatric bone disorders. Curr Osteoporos Rep. 2017;15:283–92. 10.1007/s11914-017-0380-1. 28643220 10.1007/s11914-017-0380-1 PMC5554707

[37] de Lange J, van den Akker HP, Scholtemeijer M. Cherubism treated with calcitonin: report of a case. J Oral Maxillofac Surg. 2007;65(8):1665–7. 10.1016/j.joms.2006.06.266. 17656300 10.1016/j.joms.2006.06.266

[38] Etoz OA, Dolanmaz D, Gunhan O. Treatment of cherubism with salmon calcitonin: a case report. Eur J Dent. 2011;5(4):486–91. 21912506 PMC3170034

[39] McLaughlin MB, Awosika AO, Jialal I. Calcitonin. [Updated 2023 Aug 17]. In: StatPearls [Internet]. Treasure Island (FL): StatPearls Publishing; 2026. Available from: https://www.ncbi.nlm.nih.gov/books/NBK537269 30725954

[40] Cailleaux PE, Porporatti AL, Cohen-Solal M, Kadlub N, Coudert AE. Pharmacological management of cherubism: a systematic review. Front Endocrinol (Lausanne). 2023;14: 1104025. 10.3389/fendo.2023.1104025. 36998472 10.3389/fendo.2023.1104025 PMC10044089

